# HPV Vaccines Among University Students: Understanding Barriers and Facilitators of Vaccine Uptake

**DOI:** 10.3390/vaccines12121385

**Published:** 2024-12-10

**Authors:** Sana Malik, K. Olivia Mock, Rose Martillotti, Giuseppina Caravella, Xicheng Zhou, Matthew Mbamelu, Kathleen H. Scarbrough

**Affiliations:** 1 School of Social Welfare, Stony Brook University, Stony Brook, NY 11794, USA; xicheng.zhou@stonybrook.edu; 2 Department of Psychology, Stony Brook University, Stony Brook, NY 11794, USA; kathryn.mock@stonybrook.edu (K.O.M.); rose.martillotti@stonybrook.edu (R.M.); 3 Department of Pediatrics, David Geffen School of Medicine at UCLA, Los Angeles, CA 90095, USA; 4 Stony Brook Cancer Center, Stony Brook Medicine, Stony Brook, NY 11794, USA; giuseppina.caravella@stonybrookmedicine.edu; 5 Department of Family, Population, and Preventative Medicine, Stony Brook Medicine, Stony Brook, NY 11794, USA; matthew.mbamelu@stonybrook.edu (M.M.); kathleen.scarbrough@stonybrookmedicine.edu (K.H.S.)

**Keywords:** HPV, vaccine, focus group research, qualitative analysis

## Abstract

Human papillomavirus (HPV) is the most common sexually transmitted infection and plays a significant role in cervical, penile, anal, vaginal, vulvar, and oropharyngeal cancers as well as non-cancerous genital warts and genital dysplasia. In the United States, there are approximately 46,000 new HPV-related cancers a year. There is an effective vaccine to prevent over 90% of these cancers and other HPV-related diseases; however, those that are aged 18–26 have the lowest vaccine rates among eligible age groups. The objective of this study was to examine student knowledge and perceptions about HPV vaccine hesitancy in university students and their notions of barriers and facilitators for HPV vaccine uptake. We aimed to identify components for an evidence-based community-oriented educational intervention to increase HPV vaccination uptake. The researchers recorded 10 focus groups featuring students from a Northeastern United States university, aged 18–26, which were analyzed using grounded theory and inductive thematic analysis to identify recurring themes. The participants mentioned many barriers and facilitators for attaining the HPV vaccine, with health literacy being prominent for both. They demonstrated some knowledge of what HPV is and ways to avoid it. They also expressed a desire for further information and felt that the way in which this information is presented to the public is vital for increasing vaccine uptake and designing future interventions. In order to increase HPV vaccination rates in the general population and overcome barriers such as family, religious, and cultural values, it is important to emphasize the link between HPV and cancer and its preventative benefits.

## 1. Background

Human papillomavirus (HPV) is the most prevalent sexually transmitted infection [1,2]. Annually, there are 13 million newly acquired HPV infections in the United States (U.S.), half of which affect persons aged 15 to 24 years [2]. HPV plays a significant role in cervical, penile, anal, vaginal, vulvar, and oropharyngeal cancers as well as non-cancerous genital warts and genital dysplasia [1]. Nearly every sexually active person in the U.S. will contract at least one strain of HPV in their lifetime and while most of these infections will remain asymptomatic and resolve within two years, some people with persistent infections may develop HPV-related cancer [1]. According to data from the Centers for Disease Control and Prevention [3] over 47,000 HPV-related cancers have been diagnosed annually from 2017 to 2021 in the U.S. [3]. Screening and treatment of HPV infections and HPV-related cancers cost an estimated USD 9.01 billion annually in direct costs [4].

The best prevention against HPV infection and subsequent benign lesions and cancer is the HPV vaccine, which the CDC estimates can prevent 92% of HPV-related cancers [5]. The HPV vaccine was first approved by the Food and Drug Administration (FDA) in 2006 for use in girls and has since expanded to prevent the infection in boys. In the U.S., the country in which this study takes place, Gardasil^®^9 is the only HPV vaccine available. Current recommendations by the Federal Advisory Committee on Immunization Practices (ACIP) state that the HPV vaccine should be administered as a two-dose series, 6–12 months apart, in children aged 9–14. If this ideal window is missed, catch-up vaccination is available for people aged 15–45 with a three-dose series, given at 0, 1–2 months, and 6 months apart [6]. The HPV vaccines are most effective if the series is completed before the first oral–vaginal–anal sexual contact and as such are routinely administered to children aged 11–12, but can be given as young as nine years [7,8]. Current recommendations include catch-up vaccination for individuals not previously vaccinated up to age 26 and shared decision making for those aged 27 to 45 years [6]. Current vaccination rates in all age groups fall short of the Healthy People 2030 goals of 80% completing the HPV-vaccine series goal [9]. Overall, the percentage of adults aged 18−26 who ever received one or more doses of HPV vaccine increased from 22.1% in 2013 to 39.9% in 2018 [10]. In 2018, only 54% of women and 27% of men ages 18–26 had initiated the vaccine series, with only 22% of this age group completing the series [10]. 

HPV infection and related diseases are a significant health issue facing university students given the nationally low HPV vaccination rates and their increased rates of sexual activity. In recent years, research has been conducted to understand how university-aged individuals begin to transition from their parents making their medical decisions to making their own decisions and, in particular, how this process may be related to how they make decisions regarding HPV vaccination [11,12,13,14,15]. Multiple studies have confirmed that the predisposing factors of low HPV knowledge, low HPV vaccine knowledge, and lack of awareness of susceptibility to HPV contribute to the low HPV vaccination rates among university students, with a larger knowledge deficit for males compared to females [11,16]. Among university students, the strongest concern identified about getting a vaccine was related to family and friends finding out if the student were to get vaccinated and concerns about safety and side effects [11,17]. There is evidence that social and peer influences (reinforcing factors) impact vaccine acceptance and that the stronger the perceived social support, the more likely men have the intent to vaccinate [13,14]. There are few qualitative and mixed-method studies evaluating a student’s knowledge and perspective about HPV, HPV vaccines, and potential barriers and facilitators, and even fewer studies asking students themselves about potential interventions that would increase HPV vaccinations [18,19,20]. A systematic review of university HPV vaccine intervention studies concluded that there are many studies demonstrating improvement in vaccination intention; however, very few interventions targeting university students have demonstrated effectiveness in increasing actual HPV vaccine uptake [21]. The aim of this study was to provide student insight on the topic of HPV vaccines, potential barriers/facilitators to vaccines, how they want to receive information, and potential intervention strategies to increase vaccine knowledge and uptake via small group focus groups.

The researchers developed a guided interview to encourage discussions regarding predisposing factors (such as experiences with providers and parental influences), enabling factors (such as locus of control, perceived barriers, and facilitators) associated with HPV vaccine hesitancy and uptake and HPV/HPV vaccine knowledge through interviewer-facilitated focus groups. The researchers also wanted to examine student perceptions about HPV vaccine hesitancy in university students and their notions of barriers and facilitators for HPV vaccine uptake among university students. In addition to understanding these factors regarding HPV vaccination, the researchers wanted to discover the best way to identify and design intervention components for future evidence-based community-oriented educational interventions for university students to increase HPV vaccination uptake. The focus groups were transcribed to enable an inductive thematic analysis of participant responses using a grounded theory approach. 

This study was conducted at a large public university in a suburb of a major city in the Northeastern U.S. The student body consists of a diverse group of over 25,000 students. Approximately 40% of the students identify as Asian, 40% identify as White, 13% identify as Hispanic/Latino, 9% identify as Black, and 10% are unknown; note that the total is over 100% due to data being collected using two-part questions [22]. The U.S. does not have a single-payer healthcare system and insurance must be individually procured [23]. Campus policy states that students must be enrolled in health insurance; if they do not have their own insurance coverage, a student plan is available.

## 2. Methods

### 2.1. Participants

Forty-two students participated in 10 focus groups held remotely via Zoom. These focus groups were held to assess knowledge and attitudes towards HPV and HPV vaccination and to gain insights from the students about how they would like to receive HPV-related information. The focus groups were led by one of three trained facilitators and an additional team member was present to assist in technical issues that may arise and to document observations during the focus groups. A semi-structured guide was utilized to conduct the focus groups. Each focus group was scheduled for 60 to 90 min including the time to obtain the consent, introduction, and set the rules for the focus groups. After consent was obtained, the recording of the focus group was initiated. The participants received a USD 40 gift card for their participation in the focus groups. The participants could withdraw from this study at any point. 

### 2.2. Sampling and Recruiting

An online Qualtrics survey to determine current COVID-19, Flu, and HPV vaccination rates among students, perceptions of these vaccines, and potential barriers and facilitators to receiving these vaccines were sent to all Stony Brook University (SBU) students aged 18 to 26 years (19,351 students) via an email sent out by Student Health Services in March of 2022. A reminder email was sent one week later. The full results of the quantitative survey will be reported separately. At the completion of the survey, students had the option to provide an email address if they were interested in being contacted to participate in a focus group discussing HPV vaccinations. Out of 797 completed surveys, 200 hundred students provided their email addresses. In Fall 2022, several emails were sent to these students with a description of the focus group format and a link to a doodle poll with dates/times that the focus groups were being held. When a student signed up for a focus group, they were sent a consent form to review and the Zoom invite. The inclusion criteria for participating in the focus groups were being between the ages of 18 and 26 years old, being a current SBU student, and consenting to participate. The focus groups were scheduled with 6–10 participants, although due to last-minute cancellations, some groups convened with fewer participants. The focus groups were intended to convene until thematic saturation was met. 

### 2.3. Data Collection

The focus groups were guided by a semi-structured, open-ended interview guide and took place via Zoom. The focus group guide included questions about three main themes including knowledge and perception of current HPV-related education and services, barriers and facilitators to vaccine coverage, and intervention design. Examples of questions included the following: In your opinion, what are the main questions, doubts, and fears about receiving the HPV vaccine among university students? How easy do you think it is to access/receive the HPV vaccine (e.g., who offers the vaccine and what is the cost)? If someone is not vaccinated, what reasons might motivate them to get vaccinated in university? Why might they choose not to get vaccinated? The focus groups were recorded via Zoom with the consent of participants. The focus groups lasted on average 60–90 min. Health-related resources and referrals were gathered for participants. A follow-up email was sent to participants with health-related information from the CDC and local resources about HPV and HPV vaccines. 

Demographic information such as age, sex assigned at birth, race/ethnicity, sexual orientation, relationship status, where they were born, and whether they lived in the U.S. between the ages of 9 and 17 was obtained via an online Qualtrics survey prior to participation in the focus groups. The survey also included questions about HPV vaccine status, provider recommendation for HPV vaccination, and HPV and HPV vaccine knowledge using a validated tool [24,25]. Sample HPV General Knowledge questions (True/False) included “HPV can cause cervical cancer”, “Men cannot get HPV”, and “HPV can be transmitted through oral sex” (Appendix A). Sample HPV Vaccination Knowledge Items (True/False) included “The HPV vaccine requires only 1 dose”, “The HPV vaccines are most effective if given to people who have never had sex”, and “You can cure HPV by getting the HPV vaccine” (Appendix B). Please refer to Apendices A and B for a full list of knowledge questions. The Stony Brook University Institutional Review Board approved this study (IRB #2021-00325) and the guidelines in the Declaration of Helsinki were upheld.

### 2.4. Data Analysis

The focus group interviews were automatically transcribed verbatim via Zoom, reviewed for accuracy, and imported into NVivo 12 software for content analysis. A grounded theory approach and inductive thematic analysis were employed in data analysis to allow a nuanced understanding of participants’ experiences and emergent themes [26]. A preliminary codebook, based upon a set of a priori themes, was created and utilized by the second and third authors. The authors conducted open coding of two focus group transcripts to develop an initial list of codes. After completing the open coding, the two researchers then conducted axial coding whereby the initial list of codes was defined, redefined, and recorded in an iterative process using the same two transcripts. 

The research team met weekly throughout each phase of the coding to discuss coding definitions and application. This constant comparative method throughout the initial coding phase ensured that coders adhered to the same application of the coding scheme. Discrepancies in codes were resolved via consensus between coders. Analyses revealed reoccurring themes across all focus groups, indicating thematic saturation. Given the qualitative nature of this study, a power analysis was not used to determine sample size as it was solely based on attaining an appropriate number of groups and participants to reach saturation. Descriptive statistics were conducted using SPSS version 21.

## 3. Results

The mean age of participants was 21.05 (SD = 2.37), the age range was 18–26, and a majority were female (76.2%), heterosexual (76.2%), non-Hispanic (85.7%), and undergraduate (71.4%) students. A majority of participants had a healthcare provider recommend the HPV vaccine (76.2%) and received at least one dose of it (71.4%). The focus group participants scored an average of 10.86 (SD = 6.10) out of 22 and 5.55 (SD = 2.94) out of 10 on HPV knowledge and HPV vaccine knowledge tests, respectively. Our demographic breakdown is similar to the demographic breakdown of the University, as discussed previously (refer to Table 1 for a detailed breakdown of the focus group demographics).

Qualitative analysis of the focus group transcripts revealed five overarching themes and minor edits have been made to responses for clarity.

### 3.1. Theme 1: Health Literacy

The participants indicated a number of factors that contributed to their knowledge or lack thereof of the HPV vaccine and healthcare information. Further discussion indicated that the majority of their knowledge came from their healthcare provider. One participant indicated the following:

“*So I first heard about it from my pediatrician, I think when I was 12 or 13, and she just recommended getting the vaccine because it prevents cervical cancer and my parents, at first they didn’t really know what the vaccine was, but my pediatrician reassured them that it’s a good vaccine to get, because it’s one of the few vaccines that prevents cancer.*”

Other sources of information were various online sources and their social circles (family and friends).

“*I have a friend who is super health conscious, and she always sends reminders to our friend group like, ‘Go get the flu vaccine or go get this vaccine.’*”

They commonly associated HPV with cancer and disease and recognized the preventative nature of the vaccine.

“*It’s a virus or the vaccine protects you against getting the virus, and I’m not sure but I’m pretty sure it causes cervical cancer, the virus.*”

Overall, the participants indicated having heard about HPV and the vaccine; however, they were not one hundred percent confident in the accuracy and comprehensiveness of their knowledge. The participants mentioned that they are unfamiliar with the vaccine and do not have much information due to minimal health literacy issues or lack of knowledge. The participants reported that low health literacy and confusion regarding HPV, the vaccine, and the vaccination process were barriers to them receiving the vaccine.

“*I decided not to fill out the form for now, because I don’t know a lot, and I don’t have enough information to make an informed decision, and it didn’t feel like I had really anyone to ask.*”

“*I honestly don’t know that much about it. I know it’s something that my doctor has asked me about in the past, but other than that, I don’t really know anything about it.*”

“*For me? It’s a shot in the dark. I don’t know much.*”

One of the major reasons for this minimal health literacy can be attributed to familial health literacy and attitudes towards vaccines in general, as well as sexual activity.

“*I would say, maybe, if your parents were against any type of vaccine that could possibly impact your way of viewing vaccines, because you haven’t seen a different view. You’ll only see what your parents taught you.*”

“*I guess she heard STIs or cervical cancers, and sexually active, and she was like, ‘No, my daughter doesn’t do that.’ So that was kind of her point of view.*”

Related to family values regarding sexual activity, religious beliefs also played a role in limited vaccine knowledge and accessibility:

“*When I used to go to a Catholic school, a lot of people took thousands of chastity and whatnot, and they would save themselves for marriage. So, they may not see a reason in receiving a vaccine.*”

In addition to family and religious beliefs, some participants felt that there were also cultural and language barriers to health literacy of the HPV vaccine:

“*I think a little bit of both, I think, culturally, we don’t really talk about illness like specific illnesses other than just a fever, common cold stuff like that. I think what makes HPV even a bit more taboo is the fact that you can get it from sexual intercourse, and that’s something that our family and I know for a lot of families don’t really talk about in their culture.*”

“*Spanish being her primary language, it was a little bit difficult with the communication of exactly what the vaccine was, and what was its purpose. And so, we did receive a pamphlet that was in the office, and we did read through it. It did take some time for my mom to finally be like, Okay, fine. So there was definitely a kind of hesitancy with it.*”

Sex education provided in schools was also a source of hampered knowledge:

“*I mean. I heard about it in health class, but it was just kind of glossed over. It was okay. It wasn’t really talked about?*”

The main determinant of students’ perceptions regarding the HPV vaccine was health literacy. Healthcare providers, friends, and the internet were the main sources of information; however, most students lacked a concrete understanding of the vaccine

### 3.2. Theme 2: Other Barriers and Facilitators

In addition to health literacy acting as a barrier or facilitator depending on whether an individual had high health literacy, participants mentioned several additional barriers to getting the HPV vaccine, including lack of transportation, issues regarding insurance, low socioeconomic status, concerns about negative effects of the vaccine, and general medical distrust.

“*People who come from a lower socioeconomic background. So I know there’s some neighborhoods where they might not have the best coverage for doctors, and they have to travel a far distance to go see a doctor, and so I can see how that could be a hindrance.*”

“*I haven’t been with my current physician a long time, but in the short time period that I’ve known her she’s been trying to push a lot, like she’s been trying to push this vaccine on me, and I think it’s because she gets paid every time she administers the vaccine. So I’m not really that trusting of that.*”

While these barriers did deter participants from receiving the vaccine, further discussion revealed that independence acts as a facilitator to encourage individuals to get vaccinated. This includes the transition to adulthood and newfound freedom in their university lifestyle. They may be more self-motivated now due to living on their own and having the ability to make their own decisions.

“*If your parents didn’t want you to get it. But you wanted to get this vaccine being at college and likely away from home and being able to kind of make your own health decisions and make them in a way that your parents wouldn’t be informed that you had made this decision, so the freedom to do that.*”

With this new independence, participants mentioned that individuals might want to access the vaccine due to a change in sexual activity.

“*I think maybe, especially since it is STI related, in college you come from a conservative household, and now you’re allowed to explore your sexuality a little bit more. Maybe you want to have that next level of protection before putting yourself in situations.*”

Individual beliefs and socioeconomic and logistical factors are additional influencers of HPV vaccination rates.

### 3.3. Theme 3: Health Information Wanted 

When asked about what health information would be useful, participants indicated an interest in learning more in general:

“*For me. I just feel like the more you know the better. So as much as I can learn about it, it’s just always good.*”

They also expressed a desire to learn about more specific aspects of HPV and the HPV vaccine, including information about the disease itself, means of prevention, vaccine effectiveness, the vaccination process, and the risks and benefits of vaccination. They mentioned that they want to learn more about anything related to the disease, such as transmission and pervasiveness:

“*I guess, how prevalent it is, how easily spreadable it is, because you know everyone gets the vaccine, but I don’t know. I feel like there’s not much information about how widespread it is.*”

In addition to wanting to know more about how it is spread, participants mentioned that they want to learn more about HPV prevention.

“*If someone was to conduct a study of the vaccine, or just an abstract of that because I feel like that kind of just really like, even in a simple manner, it doesn’t have to be like a scientific term like nothing too complicated something that like we can understand easily, like what it’s for who it’s used for.*”

Participants further expanded on the desire to know more about prevention to focus specifically on information regarding vaccine efficacy, how successful the vaccine is in preventing the contraction of HPV, and whether efficacy wears off over time.

“*Yeah, also short term and long-term effects and just how long it lasts, and why it’s not really recommended to older people. I would just like to know more about it in general.*”

“*I’ve heard around that on the HPV vaccine, there are many variants of HPV. And of course, the vaccine can’t cover every single one, so I guess I would want to know which ones do they cover? Do they like target specific variants, and I guess how effective is the vaccine against like, I guess all of the other ones like, how much protection am I getting?*”

Participants also had questions about the HPV vaccination process in particular regarding cost, availability, and number and spacing of doses required:

“*Do you need to renew the HPV vaccine? I know I I went in, I had to get a new one. They recommended the new Tetanus shots because it’s been over 10 years. I don’t know. They told me it’s okay or I should, but I shouldn’t. If I didn’t want to get the next 10, I’m not sure if it would matter.*”

“*I wanted to ask if the HPV vaccine is available, like free to everyone, or if you need insurance to get the vaccine shot. Because if it’s not covered by insurance, and you need insurance to have that covered, then I guess that would be one of the ways that it’s not as accessible to people. Because I know that insurance, like medical insurance especially, is really really expensive, and that could prevent someone, especially in our age group, from getting that vaccine.*”

When deciding on whether or not to get vaccinated, participants mentioned that they would want to know of any potential risks or side effects associated with the HPV vaccine.

“*So for me, I would want to know the side effects from the vaccine. First, because I know there’s certain vaccines that have more severe side effects than others.*”

Finally, participants mentioned that the additional information about the health benefits of the vaccine would facilitate their decision to get vaccinated or not:

“*How severely will it impact my health to choose not to get enough? If I don’t get it, will I regret not having gotten it?*”

Participants wanted detailed, jargon-free explanations of HPV transmission and the vaccine’s benefits, efficacy, and side effects.

### 3.4. Theme 4: Ways to Avoid HPV

When prompted to identify ways to avoid contracting HPV, the most commonly reported method was safe sex, including condom usage and knowing the vaccination or HPV status of a sexual partner:

“*I guess they use protection, or just like regular check-ups to your colleges’, or your primary care physician to make sure you haven’t contracted anything.*”

“*Using protection like the condom, or something like that and then getting tested regularly, and your partner as well.*”

The second most commonly reported method of avoiding HPV was abstinence:

“*I would probably say, the one that you’d hear would be abstinence 100% protects everything.*”

“*I think the extreme we always say, is like as long as you don’t do it. There is no HPV like infections.*”

The HPV vaccine was mentioned the least as a method of avoiding HPV:

“*Yeah, I agree, like literally just getting the vaccine and protecting yourself.*”

“*I mean the vaccine is good.*”

When discussing preventive measures, students commonly referenced safe sex practices, regular health checks, and abstinence, but noted the vaccine was the primary method.

### 3.5. Theme 5: Intervention Design

The researchers asked participants what types of outreach and intervention from the university would students be receptive to and what would ideally improve vaccination rates amongst the student population. The participants wanted more information about the vaccine and felt that variety in the way in which this information was presented was important to reach more students:

“*If you provide information about how it can help deter certain cancers, and how if you’re in a relationship, that if you don’t know if the other person has it, and they didn’t get tested, it is important to get it, so just providing that information and the efficiency, and then the side effects as well.*”

“*It probably is beneficial to have different kinds of formats because sometimes you just want to read a little thing on your own, and it takes 5 min, and you get your information. But sometimes, maybe you’re the kind of person who does have a lot of questions, and you want something more interactive.*”

Specific suggestions for types of outreach included campus events or pop-ups and a variety of incentives for students:

“*I think a tabling event. It’d be nice to have fellow students drawing other students in, and then also having a healthcare professional there, just to answer any specific questions that the students wouldn’t know.*”

“*I mean, aside from my point, that I mentioned earlier about more awareness about the risks of HPV contraction, I would say give somebody a free t-shirt or a drawstring backpack after they get vaccinated and there’d be zero cases of HPV on campus.*”

In regard to the use of social media to propagate information, students were evenly divided in their endorsement of this as well as cautioning against it:

“*I think it would be effective. But then it has to be like an Instagram account where a lot of people already follow, like USG or student engagement. Then, more people would see it.*”

“*Personally, I’m not a big social media person, but when I do go on social media, I’m kind of really sus of whatever anyone says, because I feel like a lot of people are sponsored. And there’s a lot of misinformation so you can’t guarantee what’s true and what’s not.*”

In summary, students were interested in getting more information about HPV and HPV vaccines and mentioned campus events, pop-ups, social media campaigns, and inclusion of incentives as the type of outreach and intervention that would be useful; however, they felt a multi-faceted approach to outreach would reach more students. 

## 4. Discussion

In general, students’ knowledge regarding both HPV and the vaccine was low, which is reflected in the health literacy and health information wanted themes within the qualitative data. This is consistent with other findings in the literature, which demonstrate low levels of knowledge among college students about HPV, its link to cancer, and the HPV vaccine [27,28]. This poor understanding of the virus and its vaccine could also explain the low prevalence of the vaccine being listed as an effective means of preventing HPV compared to safe sex and abstinence. Despite scoring low on the knowledge tests, approximately 70% of participants had received one dose of the vaccine and over three quarters reported being told about the vaccine by their healthcare provider, indicating a willingness to listen to doctor recommendations, which conflicts with the reports of medical distrust occurring within the qualitative data. Similarly, Karki et al. (2022) and Albright et al. (2018) found that knowledge about HPV did not necessarily correlate with vaccine uptake behavior [27,29].

The most prominent theme that arose was health literacy, which served as both a barrier and facilitator among many participants. Having a high health literacy allowed participants to have more health-related knowledge, which in turn enabled them to make informed decisions about receiving the HPV vaccine. This knowledge came from a multitude of sources including online sources, healthcare providers, and social circles. Social circles in particular were made up of family members who worked in healthcare as well as health-conscious friends. They were also familiar with the associations between HPV and cancer, which has often served as a motivator to receive the vaccine [30,31]. In similar studies, addressing health literacy misconceptions and knowledge gaps has been highlighted as a key strategy to increase HPV vaccine uptake among college students [27,28]. 

On the other hand, several participants indicated low health literacy and, while they had a vague understanding of what the HPV vaccine was for and what the process of getting vaccinated entailed, as well as HPV’s association with cancer, they were unclear on the specifics and indicated that it was a source of hesitancy in getting vaccinated, which was in support of previous research [27]. Reasons for this lack of health literacy were family values, religion, culture, language barriers, and education. Family values towards sex and vaccines were the most highly cited source of low health literacy, demonstrating the importance of linking the HPV vaccine to cancer prevention in promoting uptake [32].

There were also barriers that arose in addition to health literacy, including finances, medical mistrust, and negative effects and safety concerns, which continue to be reasons for the lack of vaccine uptake even after years of HPV vaccine implementation [31,33]. Financial concerns could be broken down into confusion regarding insurance, low socioeconomic status, as well as lack of transportation. The participants indicated that they were unaware of how much the vaccine cost, if it was covered by insurance, and if insurance was even required to get the vaccine. In that same vein, those without insurance were unsure if they would be able to afford the vaccine or even afford transportation to a facility that offered it. The participants also reported distrust in “Big Pharma” and the agenda healthcare providers may have in pressuring individuals to receive the vaccine and were worried about potential immediate side effects as well as long-term health consequences. 

Although participants mentioned many barriers to receiving the vaccine, there were also facilitators specific to the population of interest that led to interest in getting vaccinated. In particular, they suggested that being away from their family, exposure to new ways of thinking, and the ability to make their own decisions could encourage people to, at the very least, seek out information on the HPV vaccine. Another facet of newfound independence associated with university life is new sexual activity, which they felt would prompt many people to seek further information as well.

The participants reported that information they were interested in receiving and would improve health literacy regarding HPV and the vaccine included more information about HPV in general, the different strains of the virus, and how it causes cancer. They also indicated a desire for a clearer explanation of the vaccination process, its effectiveness, and how it works to prevent HPV. They further specified that they would appreciate more information about the specific health benefits of getting vaccinated and what risks were present. Although low health literacy serves as a major barrier to HPV vaccine uptake, it is apparent that there is a strong desire for knowledge and willingness to seek information, which has been true for male students in previous research [17]. 

When asked about ways to avoid HPV, the most commonly mentioned method was engaging in safe sex practices, meaning condom usage, knowing a partner’s STI status, and undergoing frequent STI testing. The participants also frequently mentioned remaining abstinent as another way to avoid getting HPV. Getting vaccinated was brought up as an effective way to avoid HPV the least out of all the methods listed, though this could be attributed to the participants interpreting the question “What are effective ways of avoiding HPV?” to mean ways other than vaccination as that had already been thoroughly discussed. However, it could also speak to their limited knowledge and health literacy.

Based on the responses from the focus groups, the researchers sought to create an intervention design in order to increase vaccine uptake, and thus the participants were asked for their input. The participants mentioned that the delivery method of the information was important to them and that various approaches were necessary to reach a wide audience. They felt as though events and incentives in particular would be effective ways to get the information out to the university. This is in contrast with previous research that established publicly posted promotional and educational materials and provider recommendations that were adequately effective in increasing vaccination coverage amongst university students [34]. There were mixed responses to the use of social media as a method of delivering healthcare information. There was a relatively even split between those who would use social media as a source of information and those who would not. This ambivalence is in part due to concerns about the reliability of the information [35], and students emphasized the importance of reliable sources.

## 5. Limitations

The focus groups included students solely from a Northeastern United States university, which has a student population comprised of nearly 50% commuters; therefore, caution must be used in generalizing these results to young adults broadly as vaccine rates and opinions vary geographically and commuter students have an augmented transition to university, which allows parental roles and influence to remain relatively unchanged [15,36]. When asked about ways to prevent HPV, vaccination was mentioned last, which could have been due to this being the main topic of the focus group. This could have also made the participants assume that vaccination was implied and thus they could have been brainstorming other ideas. 

Another limitation is the low proportion of male respondents, 10 vs. 32 females. Efforts were made to recruit and include males as their perspective is vital to this work. Lower turnout rates were not surprising given that men are historically less likely to participate in research and healthcare-related activities. A systematic review performed by Borg et al. (2024) looked at barriers to male participation in healthcare research [37]. They highlight social constructs such as masculinity can be attributed to the challenges around engaging men in this type of work. While this is a limitation in our study, it is consistent with prior HPV vaccination research among university students. 

## 6. Conclusions

As HPV is the most prevalent STI, ensuring individuals receive a complete round of vaccinations is vital to reducing rates of new infections in young adults. To this end, general information about HPV and the vaccine needs to be made accessible to the public through a wide range of modalities and languages. It is particularly important to inform individuals about the vaccination process, its effectiveness, and risks and benefits. Emphasizing HPV’s links to cancer and the disease-preventative aspect of the vaccine over the status of HPV as an STI could work to minimize family, religious, and cultural values as barriers to health literacy about HPV and getting vaccinated against HPV.

## Figures and Tables

**Table 1:** Focus group demographics.

Variable	n (%), N = 42	M (SD)
**Age**	--	21.05 (2.37)
**HPV knowledge score**	--	10.86 (6.10)
**HPV vaccine knowledge score**	--	5.55 (2.94)
**Sex assigned at birth**		
Female	32 (76.2)	
Male	10 (23.8)	
**Race**		
White	15 (35.7%)	
Asian	14 (33.3%)	
Black	4 (9.5%)	
Bi/Multiracial	3 (7.1%)	
Other	4 (9.5%)	
**Hispanic**		
Yes	6 (14.3%)	
No	38 (85.7%)	
**Born in the U.S.**		
Yes	33 (78.6%)	
No	9 (21.4%)	
**Married/Long-term Relationship**		
Yes	13 (31%)	
No	29 (69%)	
**Sexual Orientation**		
Heterosexual	32 (76.2%)	
Homosexual	3 (7.1%)	
Bisexual	5 (11.9%)	
Other	2 (4.8%)	
**Transgender**		
Yes	1 (2.4%)	
No	41 (97.6%)	
**Type of Student**		
Undergraduate	30 (71.4%)	
Graduate	12 (28.6%)	
**Lived in the U.S between the ages of 9 and 17**		
Yes	37 (88.1%)	
No	5 (11.9%)	
**Received at least 1 HPV vaccine dose**		
Yes	30 (71.4%)	
No	7 (16.7%)	
Do not know	5 (4.8%)	
**Provider recommended HPV vaccine**		
Yes	32 (76.2%)	
No	4 (9.5%)	
Do not know	6 (14.3%)	

## Data Availability

The data are available upon request.

## Appendix A

**Table A1:** HPV General Knowledge Items ^1^.

	Statements (Correct Answers)
1.	HPV is very rare (F)
2.	HPV always has visible signs or symptoms (F)
3.	HPV can cause cervical cancer (T)
4.	HPV can be transmitted through genital skin-to-skin contact (T)
5.	There are many types of HPV (T)
6.	HPV can be passed on during sexual intercourse (T)
7.	HPV can cause genital warts (T)
8.	Men cannot get HPV (F)
9.	Using condoms reduces the chances of HPV transmission (T)
10.	HPV can be cured with antibiotics (F)
11.	Having many sexual partners increases the risk of HPV (T)
12.	Most sexually active people will get HPV at some point in their lives (T)
13.	A person could have HPV for many years without knowing it (T)
14.	Having sex at an early age increases the risk of getting HPV (T)
15.	HPV can cause anal cancer (T)
16.	HPV is a bacterial infection (F)
17.	HPV can be transmitted through oral sex (T)
18.	HPV can cause cancer of the penis (T)
19.	HPV can be transmitted through anal sex (T)
20.	HPV infections always lead to health problems (F)
21.	HPV can cause oral cancer (T)
22.	A person with no symptoms cannot transmit the HPV infection (F)

^1^ Items 1–15 are from Waller et al.’s scale. [25] Items 16–22 were added in Perez et al.’s study [24]. Response options are: *True, False, Don’t know.*

## Appendix B

**Table A2:** HPV Vaccination Knowledge Items ^1^.

	Statements (Correct Answer)
1.	The HPV vaccine requires only 1 dose (F)
2.	The HPV vaccines offer protection against all sexually transmitted infections (F)
3.	The HPV vaccines are most effective if given to people who have never had sex (T)
4.	Someone who has had the HPV vaccine cannot develop cervical cancer (F)
5.	The HPV vaccines offer protection against most cervical cancers (T)
6.	The HPV vaccines offer protection against genital warts (T)
7.	Girls who have had the HPV vaccine do not need a Pep test when they are older (F)
8.	The HPV vaccine protects you from every type of HPV (F)
9.	You can cure HPV by getting the HPV vaccine (F)
10.	The HPV vaccine is recommended by the CDC for both males and females aged 9 to 26 (T)

^1^ Items 1–7 are from Waller et al.’s scale [25]. Items 8–10 were added in Perez et al.’s study [24]. Item 10 was adapted to the current guidelines in the U.S. Response options are: *True, False, Don’t know.*
